# Supplementary material for: The association of DNA methylation with body mass index: distinguishing between predictors and biomarkers
Source: Clin Epigenetics. 2020 Mar 30;12:50. doi: 10.1186/s13148-020-00841-5 (PMC7106582; doi:10.1186/s13148-020-00841-5)

**Additional file 1**

**Supplementary table 1**. Table of correlations between all individual CpG sites across the different time points and between matched children (cord) and mothers (antenatal).

| **CpG** | **Weights** | **Correlations** | | | | |
| --- | --- | --- | --- | --- | --- | --- |
|  |  | **Cord and Age 7** | **Cord and Age 15** | **Age 7 and Age 15** | **Antenatal and Middle age** | **Children cord and Mothers antenatal** |
| **cg00108715** | 0.007 | 0.05 | 0.02 | 0.07 | 0.15 | 0.01 |
| **cg00222799** | 0.007 | 0.17 | 0.11 | 0.22 | 0.29 | 0.09 |
| **cg00574958** | -0.02 | -0.02 | -0.02 | 0.10 | -0.01 | -0.01 |
| **cg01130991** | 0.01 | 0.11 | 0.005 | 0.11 | 0.10 | 0.10 |
| **cg01243823** | -0.008 | 0.17 | 0.16 | 0.52 | 0.49 | 0.02 |
| **cg01368219** | 0.008 | 0.11 | 0.07 | 0.11 | 0.19 | 0.06 |
| **cg01455178** | 0.009 | 0.12 | 0.08 | 0.28 | 0.21 | 0.09 |
| **cg01526748** | 0.009 | 0.14 | 0.10 | 0.23 | 0.43 | 0.09 |
| **cg01597398** | -0.006 | 0.27 | 0.21 | 0.23 | 0.24 | 0.05 |
| **cg01671681** | -0.008 | 0.04 | 0.04 | 0.19 | 0.24 | 0.002 |
| **cg01751802** | 0.008 | 0.07 | 0.14 | 0.26 | 0.34 | 0.01 |
| **cg01798813** | 0.01 | 0.17 | 0.07 | 0.17 | 0.15 | 0.08 |
| **cg01881899** | 0.01 | -0.004 | 0.001 | 0.07 | 0.05 | 0.02 |
| **cg02286155** | 0.006 | 0.16 | 0.04 | 0.14 | 0.10 | 0.08 |
| **cg02571142** | 0.008 | 0.16 | 0.19 | 0.33 | 0.36 | 0.08 |
| **cg02711608** | -0.008 | 0.23 | 0.18 | 0.28 | 0.23 | 0.09 |
| **cg02716826** | -0.008 | 0.09 | 0.10 | 0.19 | 0.20 | 0.08 |
| **cg03078551** | -0.01 | 0.02 | 0.06 | 0.10 | 0.03 | 0.05 |
| **cg03218374** | 0.009 | 0.05 | 0.05 | 0.25 | 0.25 | 0.05 |
| **cg03500056** | 0.006 | 0.11 | 0.10 | 0.33 | 0.31 | 0.09 |
| **cg03682690** | 0.01 | 0.09 | 0.06 | 0.24 | 0.26 | 0.05 |
| **cg03725309** | -0.01 | 0.13 | 0.13 | 0.20 | 0.18 | 0.06 |
| **cg04011474** | -0.008 | 0.05 | 0.08 | 0.05 | 0.23 | 0.01 |
| **cg04286697** | 0.007 | 0.07 | 0.04 | 0.13 | 0.17 | 0.03 |
| **cg04483863** | 0.009 | 0.09 | 0.07 | 0.05 | 0.11 | 0.05 |
| **cg04557677** | -0.008 | 0.02 | 0.05 | 0.21 | 0.06 | -0.002 |
| **cg04816311** | 0.01 | 0.08 | 0.12 | 0.18 | 0.26 | 0.07 |
| **cg04927537** | 0.01 | 0.25 | 0.22 | 0.46 | 0.45 | 0.18 |
| **cg05119988** | -0.009 | 0.05 | 0.11 | 0.16 | 0.12 | 0.05 |
| **cg06192883** | 0.008 | 0.08 | 0.10 | 0.15 | 0.20 | 0.02 |
| **cg06397161** | 0.01 | 0.14 | 0.15 | 0.35 | 0.35 | 0.08 |
| **cg06500161** | 0.02 | 0.10 | 0.06 | 0.20 | 0.15 | 0.11 |
| **cg06690548** | -0.02 | 0.09 | 0.07 | 0.12 | 0.10 | 0.02 |
| **cg06876354** | 0.006 | 0.08 | 0.13 | 0.17 | 0.19 | 0.16 |
| **cg06946797** | -0.009 | 0.20 | 0.19 | 0.33 | 0.30 | 0.10 |
| **cg07012687** | 0.008 | 0.09 | 0.16 | 0.28 | 0.21 | 0.12 |
| **cg07021906** | 0.008 | 0.06 | 0.13 | 0.28 | 0.24 | 0.07 |
| **cg07037944** | -0.007 | 0.24 | 0.23 | 0.27 | 0.18 | 0.09 |
| **cg07094298** | -0.01 | 0.16 | 0.16 | 0.26 | 0.27 | 0.11 |
| **cg07504977** | 0.01 | 0.12 | 0.11 | 0.22 | 0.15 | 0.09 |
| **cg07573872** | -0.009 | 0.20 | 0.13 | 0.26 | 0.23 | 0.12 |
| **cg07730360** | 0.007 | 0.15 | 0.05 | 0.09 | 0.08 | 0.04 |
| **cg07814318** | 0.008 | 0.15 | 0.20 | 0.20 | 0.20 | 0.20 |
| **cg07955474** | -0.008 | 0.26 | 0.27 | 0.28 | 0.41 | 0.14 |
| **cg07960624** | -0.01 | 0.29 | 0.27 | 0.32 | 0.39 | 0.13 |
| **cg08548559** | -0.008 | 0.19 | 0.19 | 0.36 | 0.38 | 0.24 |
| **cg08639339** | 0.007 | 0.26 | 0.32 | 0.46 | 0.42 | 0.15 |
| **cg08857797** | 0.008 | 0.17 | 0.19 | 0.24 | 0.23 | 0.10 |
| **cg09182678** | -0.008 | 0.21 | 0.22 | 0.24 | 0.26 | 0.11 |
| **cg09349128** | -0.007 | 0.10 | 0.08 | 0.16 | 0.16 | 0.07 |
| **cg09491962** | -0.006 | 0.15 | 0.09 | 0.20 | 0.13 | 0.08 |
| **cg09607047** | 0.006 | 0.06 | -0.001 | 0.13 | 0.09 | 0.04 |
| **cg09664445** | 0.006 | 0.15 | 0.09 | 0.13 | 0.18 | 0.09 |
| **cg10092518** | -0.009 | 0.04 | -0.01 | 0.11 | 0.10 | -0.03 |
| **cg10179300** | 0.01 | 0.15 | 0.15 | 0.33 | 0.26 | 0.03 |
| **cg10192877** | 0.005 | 0.12 | 0.06 | 0.05 | 0.08 | 0.04 |
| **cg10474597** | 0.01 | 0.07 | 0.04 | 0.12 | 0.07 | 0.04 |
| **cg10508317** | -0.01 | 0.02 | -0.04 | 0.16 | 0.15 | 0.07 |
| **cg10717869** | 0.008 | 0.11 | 0.08 | 0.21 | 0.24 | 0.08 |
| **cg10919522** | -0.008 | 0.17 | 0.20 | 0.27 | 0.16 | 0.10 |
| **cg11024682** | 0.01 | 0.18 | 0.22 | 0.24 | 0.31 | 0.08 |
| **cg11152384** | -0.01 | 0.29 | 0.25 | 0.38 | 0.41 | 0.12 |
| **cg11202345** | 0.01 | 0.14 | 0.08 | 0.19 | 0.17 | 0.14 |
| **cg11261850** | 0.01 | 0.19 | 0.22 | 0.32 | 0.33 | 0.07 |
| **cg11673687** | 0.007 | 0.02 | -0.01 | 0.05 | 0.02 | 0.03 |
| **cg12001357** | -0.008 | 0.24 | 0.21 | 0.45 | 0.42 | 0.13 |
| **cg12458003** | 0.007 | 0.12 | 0.09 | 0.22 | 0.20 | 0.05 |
| **cg12484113** | 0.006 | 0.09 | 0.09 | 0.12 | 0.11 | 0.08 |
| **cg12593793** | -0.007 | 0.20 | 0.14 | 0.24 | 0.18 | 0.10 |
| **cg13028635** | 0.005 | 0.13 | 0.12 | 0.19 | 0.20 | 0.08 |
| **cg13123009** | 0.006 | 0.18 | 0.17 | 0.26 | 0.26 | 0.13 |
| **cg13134297** | -0.009 | 0.14 | 0.09 | 0.12 | 0.11 | 0.10 |
| **cg13139542** | 0.008 | 0.39 | 0.31 | 0.39 | 0.52 | 0.29 |
| **cg13274938** | 0.006 | 0.14 | 0.10 | 0.13 | 0.11 | 0.06 |
| **cg13276570** | 0.01 | 0.002 | -0.01 | 0.10 | 0.16 | 0.004 |
| **cg13305415** | 0.01 | 0.17 | 0.17 | 0.17 | 0.17 | -0.003 |
| **cg13708645** | 0.007 | 0.31 | 0.34 | 0.54 | 0.50 | 0.15 |
| **cg14017402** | 0.008 | 0.46 | 0.46 | 0.58 | 0.37 | 0.20 |
| **cg14333542** | 0.007 | 0.07 | 0.05 | 0.12 | 0.15 | 0.02 |
| **cg14352682** | 0.006 | 0.14 | 0.05 | 0.10 | 0.05 | -0.0004 |
| **cg14476101** | -0.01 | 0.42 | 0.40 | 0.50 | 0.39 | 0.18 |
| **cg14509967** | 0.01 | 0.02 | -0.06 | 0.22 | 0.19 | 0.05 |
| **cg14780837** | 0.009 | 0.11 | 0.03 | 0.14 | 0.14 | 0.004 |
| **cg14870271** | 0.01 | 0.16 | 0.16 | 0.22 | 0.25 | 0.14 |
| **cg15880704** | 0.006 | 0.16 | 0.18 | 0.22 | 0.21 | 0.11 |
| **cg16611584** | 0.01 | 0.40 | 0.38 | 0.55 | 0.59 | 0.15 |
| **cg16739178** | 0.007 | 0.13 | 0.10 | 0.16 | 0.15 | 0.05 |
| **cg17058475** | -0.01 | 0.04 | 0.04 | 0.11 | 0.09 | 0.004 |
| **cg17194270** | 0.009 | 0.20 | 0.14 | 0.23 | 0.26 | 0.12 |
| **cg17501210** | -0.01 | 0.04 | 0.02 | 0.24 | 0.37 | 0.03 |
| **cg17641710** | 0.006 | 0.09 | 0.09 | 0.08 | 0.10 | 0.05 |
| **cg17738521** | -0.01 | 0.18 | 0.10 | 0.07 | 0.14 | 0.09 |
| **cg17782974** | 0.01 | 0.10 | 0.17 | 0.16 | 0.22 | 0.13 |
| **cg17836612** | 0.009 | 0.20 | 0.18 | 0.34 | 0.30 | 0.09 |
| **cg17901584** | -0.01 | 0.18 | 0.16 | 0.27 | 0.17 | 0.11 |
| **cg18091083** | 0.01 | 0.10 | 0.03 | 0.43 | 0.54 | -0.02 |
| **cg18098839** | -0.01 | 0.10 | 0.03 | 0.09 | 0.04 | -0.03 |
| **cg18181703** | -0.007 | 0.12 | 0.04 | 0.29 | 0.27 | 0.01 |
| **cg18772573** | 0.008 | 0.16 | 0.16 | 0.17 | 0.16 | 0.13 |
| **cg19017142** | -0.01 | 0.04 | 0.04 | 0.13 | 0.16 | 0.03 |
| **cg19750657** | 0.01 | 0.15 | 0.08 | 0.23 | 0.19 | 0.11 |
| **cg20496314** | 0.009 | 0.22 | 0.20 | 0.28 | 0.18 | 0.07 |
| **cg20507228** | 0.009 | 0.07 | 0.04 | 0.21 | 0.25 | 0.02 |
| **cg21429551** | -0.01 | 0.38 | 0.36 | 0.51 | 0.47 | 0.11 |
| **cg21670987** | -0.01 | 0.29 | 0.27 | 0.46 | 0.58 | 0.10 |
| **cg21766592** | -0.01 | 0.15 | 0.16 | 0.25 | 0.19 | 0.07 |
| **cg22012981** | 0.007 | 0.07 | 0.08 | 0.03 | 0.13 | 0.04 |
| **cg22650271** | 0.008 | 0.04 | -0.02 | 0.07 | 0.12 | 0.02 |
| **cg22713958** | 0.007 | 0.18 | 0.15 | 0.30 | 0.23 | 0.11 |
| **cg22875823** | 0.009 | 0.22 | 0.24 | 0.33 | 0.29 | 0.12 |
| **cg22950899** | 0.009 | 0.15 | 0.13 | 0.24 | 0.30 | 0.06 |
| **cg23172671** | 0.009 | 0.01 | 0.10 | 0.11 | 0.19 | -0.002 |
| **cg23813257** | -0.006 | 0.30 | 0.31 | 0.35 | 0.33 | 0.14 |
| **cg23998749** | 0.007 | 0.14 | 0.07 | 0.09 | 0.18 | 0.05 |
| **cg24145109** | 0.01 | 0.45 | 0.42 | 0.59 | 0.51 | 0.23 |
| **cg24531955** | -0.009 | 0.26 | 0.23 | 0.30 | 0.28 | 0.19 |
| **cg24678869** | 0.006 | 0.08 | 0.08 | 0.13 | 0.19 | 0.04 |
| **cg25178683** | 0.009 | 0.21 | 0.22 | 0.36 | 0.35 | 0.14 |
| **cg25217710** | 0.006 | 0.14 | 0.16 | 0.18 | 0.21 | 0.07 |
| **cg25392060** | 0.006 | 0.25 | 0.20 | 0.23 | 0.25 | 0.14 |
| **cg25649826** | 0.007 | 0.22 | 0.15 | 0.18 | 0.20 | 0.14 |
| **cg26191447** | -0.01 | 0.27 | 0.25 | 0.34 | 0.53 | 0.09 |
| **cg26361535** | 0.01 | 0.29 | 0.15 | 0.30 | 0.37 | 0.04 |
| **cg26403843** | 0.01 | 0.43 | 0.39 | 0.48 | 0.47 | 0.22 |
| **cg26470501** | -0.008 | 0.27 | 0.28 | 0.33 | 0.32 | 0.18 |
| **cg26651978** | -0.007 | 0.09 | 0.05 | 0.19 | 0.24 | 0.02 |
| **cg26800893** | -0.008 | 0.10 | 0.06 | 0.09 | 0.03 | 0.01 |
| **cg26894079** | -0.009 | 0.13 | 0.16 | 0.27 | 0.28 | 0.05 |
| **cg26950531** | -0.01 | 0.14 | 0.07 | 0.15 | 0.17 | 0.03 |
| **cg26955383** | 0.007 | 0.10 | 0.11 | 0.14 | 0.22 | 0.11 |
| **cg27115863** | -0.01 | 0.24 | 0.19 | 0.34 | 0.32 | 0.07 |
| **cg27243685** | 0.01 | 0.06 | 0.07 | 0.10 | 0.14 | 0.06 |
| **cg27394566** | 0.009 | 0.17 | 0.19 | 0.23 | 0.23 | 0.10 |
| **cg27470213** | -0.01 | 0.13 | 0.07 | 0.26 | 0.18 | 0.09 |
| **cg27637521** | -0.01 | -0.03 | -0.06 | 0.06 | 0.09 | -0.02 |

**Supplementary table 2**. Table of Mendelian Randomisation type analyses to assess whether there is a causal association from BMI to each of the 135 CpG sites used to construct the methylation score.

| **CpG** | **Estimate (Lower CI, Upper CI, P-value)** | | | | |
| --- | --- | --- | --- | --- | --- |
|  | **Cord** | **Childhood** | **Adolescence** | **Middle-age** | **Antenatal** |
| **cg01455178** | -0.02 (-0.08, 0.05, p=0.61) | 0.01 (-0.05, 0.08, p=0.72) | 0.02 (-0.04, 0.09, p=0.45) | -0.03 (-0.1, 0.03, p=0.35) | -0.03 (-0.1, 0.04, p=0.4) |
| **cg03725309** | -0.002 (-0.07, 0.07, p=0.95) | -0.05 (-0.11, 0.02, p=0.14) | 0.01 (-0.06, 0.07, p=0.8) | -0.003 (-0.07, 0.06, p=0.93) | 0.04 (-0.03, 0.1, p=0.28) |
| **cg08639339** | -0.01 (-0.07, 0.06, p=0.88) | -0.04 (-0.1, 0.03, p=0.29) | -0.01 (-0.08, 0.05, p=0.73) | -0.04 (-0.1, 0.03, p=0.27) | -0.02 (-0.09, 0.05, p=0.57) |
| **cg10092518** | -0.02 (-0.08, 0.05, p=0.64) | -0.02 (-0.08, 0.05, p=0.65) | -0.02 (-0.08, 0.05, p=0.57) | -0.04 (-0.1, 0.03, p=0.28) | -0.005 (-0.07, 0.06, p=0.89) |
| **cg10717869** | 0.004 (-0.06, 0.07, p=0.91) | -0.03 (-0.1, 0.03, p=0.35) | 0.01 (-0.06, 0.07, p=0.84) | 0.03 (-0.04, 0.09, p=0.41) | -0.04 (-0.11, 0.03, p=0.22) |
| **cg11673687** | 0.03 (-0.04, 0.1, p=0.36) | 0.03 (-0.03, 0.1, p=0.32) | 0.02 (-0.04, 0.09, p=0.53) | -0.06 (-0.12, 0.01, p=0.1) | 0.005 (-0.06, 0.07, p=0.89) |
| **cg12458003** | 0.07 (-0.0004, 0.13, p=0.05) | -0.002 (-0.07, 0.06, p=0.94) | 0.01 (-0.05, 0.08, p=0.68) | 0.002 (-0.06, 0.07, p=0.94) | 0.04 (-0.03, 0.1, p=0.3) |
| **cg12484113** | -0.03 (-0.09, 0.04, p=0.44) | -0.04 (-0.11, 0.02, p=0.22) | -0.03 (-0.1, 0.03, p=0.34) | 0.04 (-0.03, 0.11, p=0.23) | 0.03 (-0.04, 0.1, p=0.35) |
| **cg12593793** | -0.04 (-0.11, 0.03, p=0.23) | -0.05 (-0.11, 0.01, p=0.13) | -0.03 (-0.1, 0.03, p=0.31) | -0.01 (-0.08, 0.06, p=0.77) | 0.02 (-0.04, 0.09, p=0.51) |
| **cg14476101** | 0.02 (-0.05, 0.08, p=0.65) | -0.01 (-0.07, 0.06, p=0.81) | -0.01 (-0.08, 0.05, p=0.7) | -0.02 (-0.08, 0.05, p=0.62) | -0.01 (-0.08, 0.06, p=0.8) |
| **cg17901584** | 0.01 (-0.06, 0.07, p=0.86) | 0.02 (-0.05, 0.08, p=0.62) | -0.01 (-0.08, 0.05, p=0.7) | 0.003 (-0.06, 0.07, p=0.93) | 0.003 (-0.06, 0.07, p=0.92) |
| **cg23172671** | -0.02 (-0.09, 0.05, p=0.52) | -0.04 (-0.1, 0.03, p=0.29) | 0.01 (-0.05, 0.08, p=0.66) | 0.04 (-0.03, 0.11, p=0.24) | 0.02 (-0.05, 0.08, p=0.63) |
| **cg23998749** | -0.02 (-0.08, 0.05, p=0.64) | -0.01 (-0.07, 0.06, p=0.84) | -0.01 (-0.08, 0.05, p=0.66) | 0.05 (-0.02, 0.11, p=0.15) | -0.05 (-0.11, 0.02, p=0.18) |
| **cg24145109** | -0.0004 (-0.07, 0.07, p=0.99) | -0.02 (-0.08, 0.05, p=0.62) | -0.005 (-0.07, 0.06, p=0.89) | 0.01 (-0.06, 0.07, p=0.83) | 0.01 (-0.06, 0.08, p=0.79) |
| **cg24678869** | -0.01 (-0.08, 0.06, p=0.76) | -0.02 (-0.08, 0.05, p=0.57) | -0.01 (-0.07, 0.06, p=0.84) | -0.02 (-0.09, 0.04, p=0.52) | 0.003 (-0.06, 0.07, p=0.92) |
| **cg25217710** | -0.03 (-0.1, 0.04, p=0.4) | -0.04 (-0.11, 0.02, p=0.18) | -0.003 (-0.07, 0.06, p=0.94) | 0.01 (-0.06, 0.07, p=0.85) | 0.01 (-0.06, 0.07, p=0.83) |
| **cg04011474** | 0.06 (-0.01, 0.13, p=0.07) | -0.05 (-0.11, 0.02, p=0.17) | -0.04 (-0.1, 0.03, p=0.26) | -0.02 (-0.08, 0.05, p=0.65) | 0.001 (-0.07, 0.07, p=0.99) |
| **cg04286697** | 0.05 (-0.02, 0.12, p=0.16) | -0.04 (-0.1, 0.03, p=0.23) | 0.01 (-0.05, 0.08, p=0.71) | -0.01 (-0.08, 0.05, p=0.71) | -0.002 (-0.07, 0.07, p=0.96) |
| **cg06876354** | 0.04 (-0.02, 0.11, p=0.2) | -0.002 (-0.07, 0.06, p=0.95) | -0.004 (-0.07, 0.06, p=0.91) | 0.05 (-0.01, 0.12, p=0.12) | 0.07 (0.005, 0.14, p=0.04) |
| **cg12001357** | -0.02 (-0.08, 0.05, p=0.66) | 0.01 (-0.05, 0.08, p=0.68) | -0.03 (-0.1, 0.03, p=0.36) | -0.002 (-0.07, 0.06, p=0.96) | -0.01 (-0.08, 0.05, p=0.72) |
| **cg13139542** | -0.04 (-0.11, 0.03, p=0.27) | -0.03 (-0.09, 0.04, p=0.39) | -0.01 (-0.08, 0.05, p=0.75) | -0.01 (-0.07, 0.06, p=0.87) | -0.01 (-0.08, 0.06, p=0.81) |
| **cg14017402** | -0.02 (-0.09, 0.04, p=0.48) | -0.05 (-0.11, 0.02, p=0.16) | -0.005 (-0.07, 0.06, p=0.89) | 0.05 (-0.02, 0.12, p=0.13) | 0.02 (-0.05, 0.08, p=0.65) |
| **cg19017142** | 0.004 (-0.06, 0.07, p=0.9) | 0.003 (-0.06, 0.07, p=0.93) | -0.03 (-0.1, 0.03, p=0.31) | -0.04 (-0.11, 0.02, p=0.22) | 0.04 (-0.03, 0.1, p=0.3) |
| **cg26191447** | -0.02 (-0.09, 0.05, p=0.53) | -0.03 (-0.1, 0.03, p=0.35) | 0.03 (-0.04, 0.09, p=0.4) | 0.05 (-0.02, 0.11, p=0.18) | 0.01 (-0.06, 0.07, p=0.84) |
| **cg00108715** | 0.06 (-0.01, 0.13, p=0.07) | -0.03 (-0.09, 0.04, p=0.43) | 0.002 (-0.06, 0.07, p=0.96) | -0.01 (-0.07, 0.06, p=0.87) | 0.06 (-0.01, 0.13, p=0.08) |
| **cg01368219** | 0.004 (-0.06, 0.07, p=0.9) | -0.01 (-0.08, 0.05, p=0.71) | 0.02 (-0.04, 0.09, p=0.51) | 0.03 (-0.03, 0.1, p=0.33) | -0.04 (-0.11, 0.03, p=0.24) |
| **cg01526748** | -0.04 (-0.1, 0.03, p=0.28) | 0.02 (-0.04, 0.09, p=0.52) | 0.04 (-0.02, 0.11, p=0.21) | 0.06 (-0.004, 0.13, p=0.07) | 0.04 (-0.03, 0.11, p=0.23) |
| **cg01671681** | 0.001 (-0.07, 0.07, p=0.98) | -0.03 (-0.09, 0.04, p=0.4) | -0.06 (-0.13, 0.001, p=0.05) | -0.03 (-0.1, 0.03, p=0.33) | -0.02 (-0.09, 0.05, p=0.59) |
| **cg07730360** | 0.02 (-0.05, 0.09, p=0.51) | -0.03 (-0.1, 0.03, p=0.33) | 0.06 (-0.001, 0.13, p=0.05) | 0.01 (-0.05, 0.08, p=0.68) | 0.05 (-0.02, 0.12, p=0.13) |
| **cg17641710** | -0.01 (-0.08, 0.05, p=0.67) | -0.01 (-0.07, 0.06, p=0.79) | -0.05 (-0.11, 0.02, p=0.17) | 0.02 (-0.05, 0.08, p=0.59) | -0.01 (-0.08, 0.06, p=0.77) |
| **cg18098839** | 0.03 (-0.04, 0.1, p=0.35) | -0.01 (-0.08, 0.05, p=0.71) | -0.04 (-0.1, 0.03, p=0.24) | 0.02 (-0.05, 0.09, p=0.56) | 0.03 (-0.04, 0.1, p=0.4) |
| **cg22012981** | 0.03 (-0.03, 0.1, p=0.34) | -0.002 (-0.07, 0.06, p=0.94) | 0.03 (-0.03, 0.1, p=0.35) | -0.004 (-0.07, 0.06, p=0.91) | -0.05 (-0.12, 0.01, p=0.12) |
| **cg05119988** | -0.01 (-0.08, 0.06, p=0.75) | 0.01 (-0.05, 0.08, p=0.66) | -0.06 (-0.12, 0.005, p=0.07) | 0.05 (-0.01, 0.12, p=0.13) | 0.003 (-0.06, 0.07, p=0.92) |
| **cg06690548** | 0.03 (-0.04, 0.09, p=0.44) | 0.04 (-0.03, 0.1, p=0.28) | 0.01 (-0.05, 0.08, p=0.7) | 0.005 (-0.06, 0.07, p=0.89) | -0.03 (-0.09, 0.04, p=0.42) |
| **cg07094298** | 0.004 (-0.06, 0.07, p=0.91) | 0.011 (-0.05, 0.08, p=0.75) | -0.02 (-0.09, 0.04, p=0.48) | 0.003 (-0.06, 0.07, p=0.94) | -0.04 (-0.1, 0.03, p=0.27) |
| **cg02286155** | 0.04 (-0.03, 0.1, p=0.3) | 0.02 (-0.04, 0.09, p=0.5) | 0.08 (0.02, 0.14, p=0.02) | -0.02 (-0.09, 0.05, p=0.53) | -0.02 (-0.09, 0.05, p=0.53) |
| **cg04483863** | -0.02 (-0.09, 0.05, p=0.56) | 0.01 (-0.06, 0.07, p=0.78) | 0.07 (0.01, 0.14, p=0.03) | -0.04 (-0.11, 0.03, p=0.22) | -0.03 (-0.1, 0.04, p=0.36) |
| **cg10179300** | -0.07 (-0.14, -0.01, p=0.03) | -0.03 (-0.09, 0.04, p=0.43) | 0.02 (-0.04, 0.09, p=0.5) | 0.02 (-0.05, 0.08, p=0.6) | -0.01 (-0.08, 0.05, p=0.68) |
| **cg13276570** | 0.01 (-0.05, 0.08, p=0.7) | -0.04 (-0.1, 0.03, p=0.25) | 0.003 (-0.06, 0.07, p=0.93) | 0.02 (-0.05, 0.09, p=0.58) | -0.07 (-0.13, -0.00003, p=0.05) |
| **cg13305415** | 0.003 (-0.06, 0.07, p=0.94) | -0.003 (-0.07, 0.06, p=0.92) | -0.02 (-0.09, 0.04, p=0.49) | -0.01 (-0.08, 0.05, p=0.7) | 0.01 (-0.06, 0.08, p=0.73) |
| **cg26403843** | -0.06 (-0.13, 0.01, p=0.08) | -0.05 (-0.11, 0.02, p=0.16) | -0.03 (-0.1, 0.03, p=0.34) | -0.001 (-0.07, 0.07, p=0.97) | -0.04 (-0.11, 0.03, p=0.24) |
| **cg13123009** | -0.05 (-0.12, 0.02, p=0.13) | -0.03 (-0.09, 0.04, p=0.42) | -0.01 (-0.07, 0.06, p=0.78) | 0.03 (-0.04, 0.09, p=0.41) | -0.01 (-0.08, 0.05, p=0.72) |
| **cg14352682** | 0.03 (-0.04, 0.09, p=0.43) | 0.004 (-0.06, 0.07, p=0.9) | -0.02 (-0.08, 0.05, p=0.64) | 0.01 (-0.06, 0.08, p=0.74) | 0.03 (-0.04, 0.1, p=0.36) |
| **cg17501210** | 0.02 (-0.05, 0.09, p=0.6) | 0.02 (-0.05, 0.08, p=0.62) | -0.02 (-0.08, 0.05, p=0.62) | -0.05 (-0.11, 0.02, p=0.17) | -0.01 (-0.08, 0.05, p=0.68) |
| **cg17738521** | -0.0002 (-0.07, 0.07, p=1) | -0.01 (-0.07, 0.06, p=0.88) | 0.03 (-0.03, 0.1, p=0.35) | 0.02 (-0.04, 0.09, p=0.49) | 0.02 (-0.05, 0.08, p=0.63) |
| **cg22875823** | 0.01 (-0.05, 0.08, p=0.71) | -0.01 (-0.08, 0.05, p=0.75) | 0.03 (-0.03, 0.1, p=0.33) | -0.01 (-0.07, 0.06, p=0.88) | 0.04 (-0.02, 0.11, p=0.21) |
| **cg04816311** | 0.05 (-0.02, 0.12, p=0.15) | 0.01 (-0.06, 0.07, p=0.79) | 0.03 (-0.03, 0.1, p=0.36) | -0.06 (-0.12, 0.01, p=0.1) | 0.004 (-0.06, 0.07, p=0.91) |
| **cg13134297** | -0.04 (-0.1, 0.03, p=0.28) | 0.02 (-0.05, 0.08, p=0.58) | -0.01 (-0.08, 0.05, p=0.71) | -0.02 (-0.09, 0.04, p=0.52) | -0.01 (-0.08, 0.05, p=0.69) |
| **cg21429551** | -0.02 (-0.09, 0.04, p=0.47) | 0.01 (-0.06, 0.07, p=0.87) | -0.03 (-0.1, 0.03, p=0.34) | 0.01 (-0.06, 0.08, p=0.8) | -0.03 (-0.09, 0.04, p=0.44) |
| **cg02571142** | -0.003 (-0.07, 0.06, p=0.94) | -0.08 (-0.14, -0.01, p=0.02) | -0.02 (-0.09, 0.04, p=0.49) | -0.03 (-0.1, 0.03, p=0.34) | 0.003 (-0.06, 0.07, p=0.93) |
| **cg07960624** | 0.05 (-0.02, 0.12, p=0.16) | -0.01 (-0.07, 0.06, p=0.79) | -0.02 (-0.09, 0.04, p=0.47) | -0.01 (-0.08, 0.05, p=0.66) | -0.01 (-0.07, 0.06, p=0.85) |
| **cg24531955** | 0.02 (-0.05, 0.09, p=0.6) | -0.01 (-0.07, 0.06, p=0.79) | -0.03 (-0.09, 0.04, p=0.39) | 0.01 (-0.06, 0.08, p=0.74) | 0.004 (-0.06, 0.07, p=0.91) |
| **cg25392060** | -0.01 (-0.08, 0.06, p=0.81) | -0.05 (-0.11, 0.02, p=0.16) | -0.03 (-0.09, 0.04, p=0.4) | -0.01 (-0.08, 0.05, p=0.66) | -0.003 (-0.07, 0.06, p=0.92) |
| **cg26361535** | 0.01 (-0.05, 0.08, p=0.71) | -0.04 (-0.1, 0.03, p=0.23) | 0.06 (-0.01, 0.12, p=0.07) | -0.02 (-0.09, 0.04, p=0.48) | -0.07 (-0.14, -0.003, p=0.04) |
| **cg02716826** | 0.02 (-0.05, 0.09, p=0.56) | -0.03 (-0.09, 0.04, p=0.41) | 0.004 (-0.06, 0.07, p=0.91) | 0.04 (-0.03, 0.11, p=0.24) | 0.004 (-0.06, 0.07, p=0.92) |
| **cg07504977** | -0.02 (-0.09, 0.04, p=0.5) | -0.08 (-0.14, -0.01, p=0.02) | -0.04 (-0.1, 0.03, p=0.28) | 0.005 (-0.06, 0.07, p=0.89) | -0.01 (-0.08, 0.05, p=0.68) |
| **cg09491962** | -0.002 (-0.07, 0.07, p=0.96) | -0.02 (-0.09, 0.04, p=0.51) | -0.02 (-0.08, 0.05, p=0.64) | -0.02 (-0.09, 0.05, p=0.55) | 0.02 (-0.05, 0.08, p=0.61) |
| **cg14333542** | 0.01 (-0.06, 0.08, p=0.74) | -0.02 (-0.09, 0.04, p=0.5) | -0.01 (-0.07, 0.06, p=0.77) | -0.07 (-0.13, -0.001, p=0.05) | 0.004 (-0.06, 0.07, p=0.9) |
| **cg15880704** | -0.01 (-0.07, 0.06, p=0.83) | 0.04 (-0.03, 0.1, p=0.23) | 0.02 (-0.05, 0.08, p=0.59) | -0.02 (-0.09, 0.04, p=0.48) | -0.05 (-0.11, 0.02, p=0.17) |
| **cg17782974** | 0.002 (-0.07, 0.07, p=0.96) | 0.01 (-0.06, 0.07, p=0.87) | -0.03 (-0.1, 0.03, p=0.31) | 0.01 (-0.05, 0.08, p=0.73) | -0.04 (-0.11, 0.03, p=0.24) |
| **cg26955383** | 0.01 (-0.05, 0.08, p=0.67) | -0.004 (-0.07, 0.06, p=0.91) | -0.01 (-0.08, 0.05, p=0.73) | 0.04 (-0.03, 0.11, p=0.25) | 0.01 (-0.06, 0.08, p=0.75) |
| **cg00574958** | -0.02 (-0.09, 0.04, p=0.51) | 0.001 (-0.06, 0.07, p=0.97) | 0.01 (-0.06, 0.07, p=0.85) | 0.02 (-0.04, 0.09, p=0.48) | 0.002 (-0.07, 0.07, p=0.95) |
| **cg11152384** | 0.05 (-0.01, 0.12, p=0.11) | -0.04 (-0.11, 0.02, p=0.21) | 0.01 (-0.06, 0.07, p=0.87) | -0.04 (-0.11, 0.02, p=0.21) | -0.02 (-0.08, 0.05, p=0.66) |
| **cg11261850** | -0.02 (-0.09, 0.05, p=0.52) | 0.02 (-0.05, 0.08, p=0.64) | -0.004 (-0.07, 0.06, p=0.9) | -0.03 (-0.09, 0.04, p=0.42) | -0.02 (-0.09, 0.04, p=0.47) |
| **cg13028635** | 0.04 (-0.03, 0.11, p=0.24) | 0.03 (-0.03, 0.1, p=0.33) | 0.05 (-0.01, 0.12, p=0.11) | -0.01 (-0.07, 0.06, p=0.88) | -0.02 (-0.09, 0.05, p=0.57) |
| **cg17058475** | 0.02 (-0.05, 0.09, p=0.55) | -0.02 (-0.09, 0.04, p=0.51) | -0.01 (-0.08, 0.05, p=0.68) | 0.01 (-0.06, 0.07, p=0.83) | 0.01 (-0.06, 0.08, p=0.79) |
| **cg26800893** | 0.003 (-0.06, 0.07, p=0.93) | -0.06 (-0.12, 0.01, p=0.09) | 0.02 (-0.04, 0.09, p=0.5) | 0.01 (-0.06, 0.07, p=0.83) | 0.03 (-0.04, 0.09, p=0.44) |
| **cg26894079** | 0.003 (-0.06, 0.07, p=0.92) | -0.01 (-0.07, 0.06, p=0.82) | 0.02 (-0.05, 0.08, p=0.61) | 0.01 (-0.06, 0.08, p=0.74) | 0.01 (-0.06, 0.08, p=0.76) |
| **cg13708645** | -0.06 (-0.13, 0.01, p=0.09) | 0.02 (-0.05, 0.08, p=0.63) | 0.02 (-0.05, 0.08, p=0.63) | -0.05 (-0.12, 0.02, p=0.13) | -0.03 (-0.09, 0.04, p=0.45) |
| **cg10474597** | -0.03 (-0.09, 0.04, p=0.44) | -0.01 (-0.07, 0.06, p=0.81) | 0.03 (-0.03, 0.1, p=0.34) | 0.004 (-0.06, 0.07, p=0.91) | 0.02 (-0.05, 0.08, p=0.65) |
| **cg19750657** | 0.01 (-0.05, 0.08, p=0.69) | 0.003 (-0.06, 0.07, p=0.92) | -0.03 (-0.09, 0.04, p=0.41) | 0.01 (-0.06, 0.08, p=0.8) | 0.04 (-0.02, 0.11, p=0.2) |
| **cg10919522** | 0.003 (-0.06, 0.07, p=0.93) | 0.01 (-0.06, 0.07, p=0.86) | -0.05 (-0.12, 0.01, p=0.12) | 0.03 (-0.04, 0.09, p=0.42) | -0.03 (-0.09, 0.04, p=0.44) |
| **cg27394566** | -0.01 (-0.08, 0.06, p=0.78) | -0.01 (-0.07, 0.06, p=0.78) | 0.05 (-0.02, 0.11, p=0.14) | -0.06 (-0.12, 0.01, p=0.09) | -0.04 (-0.11, 0.03, p=0.22) |
| **cg06192883** | -0.02 (-0.09, 0.05, p=0.56) | -0.02 (-0.08, 0.05, p=0.59) | -0.005 (-0.07, 0.06, p=0.89) | 0.06 (-0.01, 0.12, p=0.1) | 0.01 (-0.06, 0.07, p=0.83) |
| **cg07037944** | 0.01 (-0.06, 0.08, p=0.77) | -0.05 (-0.12, 0.01, p=0.12) | -0.02 (-0.09, 0.04, p=0.49) | 0.02 (-0.05, 0.08, p=0.63) | 0.03 (-0.04, 0.1, p=0.38) |
| **cg07814318** | -0.05 (-0.12, 0.02, p=0.14) | -0.003 (-0.07, 0.06, p=0.92) | 0.02 (-0.04, 0.09, p=0.52) | -0.01 (-0.08, 0.06, p=0.73) | -0.003 (-0.07, 0.06, p=0.93) |
| **cg20507228** | -0.01 (-0.08, 0.06, p=0.72) | 0.01 (-0.05, 0.08, p=0.75) | -0.02 (-0.08, 0.05, p=0.63) | 0.01 (-0.05, 0.08, p=0.7) | -0.004 (-0.07, 0.06, p=0.9) |
| **cg21670987** | 0.03 (-0.03, 0.1, p=0.33) | 0.05 (-0.01, 0.12, p=0.12) | 0.01 (-0.05, 0.08, p=0.7) | -0.02 (-0.08, 0.05, p=0.63) | 0.002 (-0.07, 0.07, p=0.95) |
| **cg01243823** | 0.07 (0.004, 0.14, p=0.04) | -0.04 (-0.11, 0.02, p=0.19) | -0.06 (-0.12, 0.01, p=0.07) | -0.02 (-0.08, 0.05, p=0.66) | -0.02 (-0.09, 0.04, p=0.5) |
| **cg03500056** | -0.03 (-0.1, 0.04, p=0.39) | 0.02 (-0.05, 0.08, p=0.61) | -0.01 (-0.07, 0.06, p=0.85) | -0.005 (-0.07, 0.06, p=0.89) | 0.02 (-0.04, 0.09, p=0.52) |
| **cg06946797** | 0.03 (-0.04, 0.09, p=0.44) | -0.02 (-0.08, 0.05, p=0.62) | -0.04 (-0.11, 0.02, p=0.21) | -0.05 (-0.12, 0.02, p=0.14) | 0.02 (-0.04, 0.09, p=0.48) |
| **cg07021906** | -0.02 (-0.09, 0.05, p=0.51) | -0.03 (-0.09, 0.04, p=0.41) | 0.03 (-0.03, 0.1, p=0.29) | 0.01 (-0.06, 0.07, p=0.82) | -0.01 (-0.07, 0.06, p=0.84) |
| **cg07955474** | -0.04 (-0.11, 0.03, p=0.26) | -0.05 (-0.12, 0.01, p=0.13) | -0.03 (-0.1, 0.03, p=0.33) | -0.01 (-0.08, 0.06, p=0.8) | -0.004 (-0.07, 0.06, p=0.91) |
| **cg09607047** | -0.04 (-0.11, 0.03, p=0.25) | 0.02 (-0.05, 0.08, p=0.62) | 0.02 (-0.05, 0.08, p=0.56) | 0.02 (-0.04, 0.09, p=0.52) | 0.01 (-0.06, 0.07, p=0.87) |
| **cg16739178** | 0.01 (-0.06, 0.08, p=0.8) | -0.01 (-0.08, 0.05, p=0.71) | 0.05 (-0.02, 0.11, p=0.17) | -0.04 (-0.11, 0.03, p=0.25) | -0.001 (-0.07, 0.07, p=0.98) |
| **cg23813257** | 0.03 (-0.04, 0.1, p=0.38) | 0.02 (-0.05, 0.08, p=0.64) | 0.01 (-0.05, 0.08, p=0.7) | 0.02 (-0.04, 0.09, p=0.47) | -0.02 (-0.08, 0.05, p=0.61) |
| **cg01130991** | -0.04 (-0.1, 0.03, p=0.3) | -0.005 (-0.07, 0.06, p=0.89) | 0.02 (-0.04, 0.09, p=0.51) | -0.02 (-0.09, 0.05, p=0.54) | -0.004 (-0.07, 0.06, p=0.91) |
| **cg01597398** | -0.005 (-0.07, 0.06, p=0.89) | -0.01 (-0.07, 0.06, p=0.85) | -0.04 (-0.11, 0.02, p=0.19) | -0.01 (-0.08, 0.05, p=0.67) | 0.01 (-0.06, 0.07, p=0.83) |
| **cg01798813** | -0.03 (-0.1, 0.04, p=0.41) | -0.01 (-0.07, 0.06, p=0.86) | 0.01 (-0.06, 0.07, p=0.78) | -0.01 (-0.08, 0.06, p=0.79) | -0.03 (-0.1, 0.04, p=0.36) |
| **cg03078551** | -0.05 (-0.11, 0.02, p=0.18) | -0.03 (-0.09, 0.04, p=0.4) | -0.03 (-0.09, 0.04, p=0.42) | -0.004 (-0.07, 0.06, p=0.9) | -0.0004 (-0.07, 0.07, p=0.99) |
| **cg04927537** | -0.03 (-0.1, 0.04, p=0.41) | -0.03 (-0.1, 0.03, p=0.31) | 0.003 (-0.06, 0.07, p=0.92) | 0.06 (-0.01, 0.12, p=0.09) | -0.004 (-0.07, 0.06, p=0.91) |
| **cg07012687** | -0.02 (-0.09, 0.04, p=0.5) | -0.02 (-0.08, 0.05, p=0.58) | -0.01 (-0.07, 0.06, p=0.81) | 0.0001 (-0.07, 0.07, p=1) | 0.03 (-0.04, 0.1, p=0.39) |
| **cg08857797** | -0.02 (-0.08, 0.05, p=0.61) | 0.03 (-0.03, 0.1, p=0.36) | 0.01 (-0.05, 0.08, p=0.68) | 0.02 (-0.05, 0.08, p=0.65) | 0.04 (-0.03, 0.1, p=0.27) |
| **cg09664445** | -0.02 (-0.09, 0.05, p=0.52) | -0.07 (-0.13, -0.001, p=0.05) | -0.06 (-0.12, 0.01, p=0.08) | 0.02 (-0.05, 0.09, p=0.56) | 0.05 (-0.01, 0.12, p=0.13) |
| **cg10508317** | 0.02 (-0.05, 0.08, p=0.62) | 0.02 (-0.04, 0.09, p=0.52) | -0.05 (-0.12, 0.01, p=0.13) | 0.02 (-0.05, 0.09, p=0.54) | 0.04 (-0.03, 0.1, p=0.29) |
| **cg11024682** | -0.08 (-0.15, -0.01, p=0.02) | -0.05 (-0.11, 0.02, p=0.13) | -0.01 (-0.07, 0.06, p=0.85) | -0.02 (-0.09, 0.05, p=0.55) | 0.02 (-0.05, 0.09, p=0.52) |
| **cg11202345** | -0.02 (-0.08, 0.05, p=0.64) | -0.05 (-0.11, 0.02, p=0.17) | -0.08 (-0.14, -0.01, p=0.02) | 0.05 (-0.01, 0.12, p=0.12) | 0.01 (-0.05, 0.08, p=0.69) |
| **cg13274938** | 0.01 (-0.06, 0.08, p=0.74) | -0.01 (-0.08, 0.05, p=0.71) | 0.003 (-0.06, 0.07, p=0.93) | 0.04 (-0.02, 0.11, p=0.19) | -0.06 (-0.12, 0.01, p=0.1) |
| **cg14509967** | -0.02 (-0.08, 0.05, p=0.63) | -0.01 (-0.08, 0.05, p=0.67) | 0.03 (-0.03, 0.1, p=0.34) | 0.04 (-0.03, 0.11, p=0.25) | 0.0004 (-0.07, 0.07, p=0.99) |
| **cg14870271** | -0.02 (-0.09, 0.04, p=0.49) | -0.05 (-0.12, 0.01, p=0.13) | -0.06 (-0.13, 0.004, p=0.07) | 0.02 (-0.05, 0.09, p=0.56) | -0.02 (-0.09, 0.05, p=0.58) |
| **cg16611584** | -0.03 (-0.1, 0.04, p=0.41) | -0.03 (-0.1, 0.03, p=0.34) | -0.03 (-0.1, 0.03, p=0.32) | -0.01 (-0.08, 0.06, p=0.74) | 0.02 (-0.05, 0.09, p=0.55) |
| **cg17836612** | 0.002 (-0.07, 0.07, p=0.96) | -0.01 (-0.07, 0.06, p=0.88) | 0.03 (-0.04, 0.09, p=0.4) | 0.00003 (-0.07, 0.07, p=1) | -0.04 (-0.11, 0.03, p=0.24) |
| **cg18091083** | 0.06 (-0.003, 0.13, p=0.06) | -0.02 (-0.08, 0.05, p=0.59) | 0.02 (-0.05, 0.08, p=0.6) | 0.05 (-0.01, 0.12, p=0.12) | 0.04 (-0.03, 0.11, p=0.24) |
| **cg18181703** | 0.05 (-0.02, 0.12, p=0.16) | -0.03 (-0.1, 0.03, p=0.31) | -0.03 (-0.09, 0.04, p=0.45) | 0.01 (-0.06, 0.08, p=0.75) | 0.004 (-0.06, 0.07, p=0.9) |
| **cg18772573** | -0.03 (-0.1, 0.04, p=0.37) | 0.07 (0.001, 0.13, p=0.05) | 0.05 (-0.01, 0.12, p=0.1) | 0.01 (-0.06, 0.08, p=0.74) | 0.02 (-0.05, 0.08, p=0.64) |
| **cg22713958** | 0.04 (-0.03, 0.1, p=0.28) | -0.03 (-0.09, 0.04, p=0.42) | 0.04 (-0.03, 0.1, p=0.23) | 0.03 (-0.03, 0.1, p=0.33) | -0.02 (-0.09, 0.05, p=0.54) |
| **cg25178683** | -0.001 (-0.07, 0.07, p=0.97) | -0.03 (-0.09, 0.04, p=0.44) | 0.01 (-0.05, 0.08, p=0.72) | 0.03 (-0.04, 0.1, p=0.37) | -0.02 (-0.08, 0.05, p=0.63) |
| **cg25649826** | 0.01 (-0.06, 0.08, p=0.74) | -0.02 (-0.08, 0.05, p=0.63) | -0.002 (-0.07, 0.06, p=0.95) | 0.03 (-0.03, 0.1, p=0.34) | -0.02 (-0.09, 0.05, p=0.58) |
| **cg26651978** | -0.01 (-0.08, 0.06, p=0.74) | -0.02 (-0.09, 0.04, p=0.48) | 0.003 (-0.06, 0.07, p=0.92) | -0.05 (-0.11, 0.02, p=0.17) | 0.02 (-0.05, 0.08, p=0.64) |
| **cg27470213** | 0.02 (-0.05, 0.08, p=0.63) | 0.02 (-0.05, 0.08, p=0.63) | -0.02 (-0.08, 0.05, p=0.55) | -0.03 (-0.1, 0.04, p=0.36) | 0.02 (-0.05, 0.08, p=0.63) |
| **cg27637521** | 0.02 (-0.05, 0.09, p=0.51) | -0.01 (-0.07, 0.06, p=0.83) | -0.05 (-0.11, 0.02, p=0.16) | 0.005 (-0.06, 0.07, p=0.89) | 0.03 (-0.03, 0.1, p=0.32) |
| **cg01751802** | -0.01 (-0.08, 0.06, p=0.79) | -0.04 (-0.1, 0.03, p=0.27) | -0.01 (-0.08, 0.05, p=0.67) | -0.05 (-0.11, 0.02, p=0.18) | -0.03 (-0.1, 0.04, p=0.39) |
| **cg02711608** | -0.02 (-0.09, 0.04, p=0.48) | -0.01 (-0.08, 0.05, p=0.67) | -0.01 (-0.08, 0.05, p=0.71) | 0.02 (-0.05, 0.09, p=0.55) | 0.001 (-0.07, 0.07, p=0.98) |
| **cg04557677** | 0.02 (-0.05, 0.08, p=0.65) | -0.03 (-0.1, 0.03, p=0.29) | -0.01 (-0.07, 0.06, p=0.86) | 0.004 (-0.06, 0.07, p=0.91) | 0.04 (-0.03, 0.11, p=0.23) |
| **cg07573872** | 0.0002 (-0.07, 0.07, p=1) | 0.01 (-0.05, 0.08, p=0.7) | -0.02 (-0.08, 0.05, p=0.57) | -0.02 (-0.08, 0.05, p=0.61) | -0.001 (-0.07, 0.07, p=0.98) |
| **cg21766592** | 0.01 (-0.06, 0.08, p=0.78) | 0.01 (-0.05, 0.08, p=0.67) | -0.07 (-0.14, -0.01, p=0.03) | -0.03 (-0.1, 0.03, p=0.35) | -0.01 (-0.08, 0.05, p=0.68) |
| **cg22950899** | -0.03 (-0.1, 0.04, p=0.4) | 0.03 (-0.04, 0.09, p=0.43) | 0.002 (-0.06, 0.07, p=0.96) | 0.03 (-0.04, 0.09, p=0.4) | -0.02 (-0.08, 0.05, p=0.62) |
| **cg26470501** | 0.004 (-0.06, 0.07, p=0.92) | -0.06 (-0.12, 0.01, p=0.09) | -0.02 (-0.08, 0.05, p=0.55) | -0.03 (-0.1, 0.03, p=0.3) | 0.004 (-0.06, 0.07, p=0.91) |
| **cg26950531** | 0.06 (-0.01, 0.13, p=0.07) | 0.03 (-0.04, 0.09, p=0.38) | 0.04 (-0.02, 0.11, p=0.19) | -0.02 (-0.08, 0.05, p=0.65) | 0.02 (-0.04, 0.09, p=0.47) |
| **cg03218374** | -0.01 (-0.08, 0.05, p=0.67) | -0.003 (-0.07, 0.06, p=0.94) | 0.03 (-0.03, 0.1, p=0.32) | -0.01 (-0.08, 0.06, p=0.8) | -0.001 (-0.07, 0.07, p=0.98) |
| **cg00222799** | 0.01 (-0.06, 0.08, p=0.77) | -0.05 (-0.12, 0.01, p=0.11) | -0.02 (-0.09, 0.04, p=0.51) | -0.04 (-0.11, 0.02, p=0.21) | -0.01 (-0.08, 0.06, p=0.8) |
| **cg01881899** | 0.01 (-0.06, 0.07, p=0.86) | -0.03 (-0.09, 0.04, p=0.38) | 0.002 (-0.06, 0.07, p=0.95) | 0.02 (-0.05, 0.09, p=0.57) | 0.06 (-0.01, 0.13, p=0.08) |
| **cg06500161** | 0.11 (0.04, 0.17, p=0.002) | -0.07 (-0.13, -0.001, p=0.05) | -0.02 (-0.09, 0.04, p=0.54) | 0.03 (-0.04, 0.1, p=0.36) | -0.04 (-0.11, 0.02, p=0.21) |
| **cg10192877** | 0.01 (-0.06, 0.08, p=0.74) | 0.001 (-0.06, 0.07, p=0.97) | 0.05 (-0.01, 0.12, p=0.13) | -0.02 (-0.09, 0.04, p=0.48) | 0.004 (-0.06, 0.07, p=0.91) |
| **cg27243685** | 0.07 (0.002, 0.14, p=0.04) | 0.02 (-0.04, 0.09, p=0.53) | -0.01 (-0.08, 0.05, p=0.74) | 0.01 (-0.06, 0.08, p=0.8) | -0.04 (-0.11, 0.03, p=0.26) |
| **cg03682690** | 0.02 (-0.05, 0.09, p=0.59) | -0.03 (-0.09, 0.04, p=0.38) | 0.01 (-0.06, 0.07, p=0.84) | -0.02 (-0.08, 0.05, p=0.64) | -0.003 (-0.07, 0.06, p=0.93) |
| **cg06397161** | 0.02 (-0.04, 0.09, p=0.48) | 0.004 (-0.06, 0.07, p=0.91) | -0.03 (-0.09, 0.04, p=0.44) | -0.003 (-0.07, 0.06, p=0.93) | 0.03 (-0.04, 0.1, p=0.36) |
| **cg08548559** | -0.02 (-0.09, 0.04, p=0.49) | -0.05 (-0.11, 0.02, p=0.15) | -0.04 (-0.1, 0.03, p=0.25) | -0.01 (-0.08, 0.05, p=0.69) | -0.004 (-0.07, 0.06, p=0.9) |
| **cg09182678** | 0.02 (-0.05, 0.09, p=0.6) | -0.002 (-0.07, 0.06, p=0.94) | -0.01 (-0.08, 0.05, p=0.76) | -0.03 (-0.1, 0.03, p=0.35) | 0.04 (-0.03, 0.1, p=0.27) |
| **cg09349128** | -0.03 (-0.1, 0.04, p=0.42) | -0.05 (-0.11, 0.02, p=0.15) | -0.03 (-0.09, 0.04, p=0.4) | -0.04 (-0.1, 0.03, p=0.29) | 0.03 (-0.03, 0.1, p=0.32) |
| **cg14780837** | 0.01 (-0.05, 0.08, p=0.71) | 0.04 (-0.03, 0.1, p=0.28) | 0.02 (-0.04, 0.09, p=0.53) | 0.01 (-0.06, 0.07, p=0.84) | -0.01 (-0.08, 0.05, p=0.68) |
| **cg17194270** | 0.02 (-0.05, 0.09, p=0.53) | -0.04 (-0.1, 0.03, p=0.24) | 0.01 (-0.06, 0.07, p=0.83) | -0.0004 (-0.07, 0.07, p=0.99) | 0.06 (-0.01, 0.13, p=0.08) |
| **cg20496314** | -0.001 (-0.07, 0.07, p=0.97) | -0.02 (-0.08, 0.05, p=0.57) | -0.04 (-0.1, 0.02, p=0.23) | -0.01 (-0.08, 0.05, p=0.67) | 0.02 (-0.04, 0.09, p=0.5) |
| **cg22650271** | -0.01 (-0.07, 0.06, p=0.84) | 0.03 (-0.04, 0.09, p=0.43) | 0.02 (-0.05, 0.08, p=0.56) | -0.06 (-0.13, 0.003, p=0.06) | 0.01 (-0.06, 0.08, p=0.77) |
| **cg27115863** | 0.01 (-0.05, 0.08, p=0.71) | -0.01 (-0.08, 0.05, p=0.75) | -0.05 (-0.11, 0.02, p=0.15) | -0.02 (-0.08, 0.05, p=0.64) | 0.02 (-0.05, 0.08, p=0.63) |

**Supplementary table 3.** Table of Mendelian Randomisation type analyses, using a mixed model combining mother and child data, to further assess whether there is a causal association from BMI to each of the 135 CpG sites used to construct the methylation score.

| **CpG** | **Estimate** | **P-value** | **Lower CI** | **Upper CI** |
| --- | --- | --- | --- | --- |
| **cg01455178** | -0.004 | 0.68 | -0.02 | 0.01 |
| **cg03725309** | 0.001 | 0.93 | -0.01 | 0.01 |
| **cg08639339** | -0.005 | 0.39 | -0.02 | 0.01 |
| **cg10092518** | -0.006 | 0.33 | -0.02 | 0.01 |
| **cg10717869** | 0.0002 | 0.98 | -0.01 | 0.01 |
| **cg11673687** | -0.001 | 0.83 | -0.01 | 0.01 |
| **cg12458003** | 0.003 | 0.70 | -0.01 | 0.02 |
| **cg12484113** | 0.003 | 0.64 | -0.01 | 0.01 |
| **cg12593793** | -0.007 | 0.23 | -0.02 | 0.004 |
| **cg14476101** | -0.004 | 0.74 | -0.03 | 0.02 |
| **cg17901584** | -0.006 | 0.57 | -0.03 | 0.02 |
| **cg23172671** | 0.01 | 0.17 | -0.01 | 0.03 |
| **cg23998749** | 0.007 | 0.36 | -0.01 | 0.02 |
| **cg24145109** | 0.002 | 0.85 | -0.02 | 0.02 |
| **cg24678869** | -0.00002 | 1.00 | -0.01 | 0.01 |
| **cg25217710** | -0.002 | 0.83 | -0.02 | 0.01 |
| **cg04011474** | -0.01 | 0.12 | -0.03 | 0.003 |
| **cg04286697** | 0.0002 | 0.98 | -0.01 | 0.01 |
| **cg06876354** | 0.006 | 0.26 | -0.005 | 0.02 |
| **cg12001357** | -0.007 | 0.41 | -0.02 | 0.01 |
| **cg13139542** | 0.0001 | 0.98 | -0.01 | 0.01 |
| **cg14017402** | 0.004 | 0.74 | -0.02 | 0.02 |
| **cg19017142** | -0.01 | 0.10 | -0.03 | 0.003 |
| **cg26191447** | 0.01 | 0.16 | -0.01 | 0.03 |
| **cg00108715** | 0.001 | 0.90 | -0.01 | 0.01 |
| **cg01368219** | 0.01 | 0.10 | -0.003 | 0.03 |
| **cg01526748** | 0.02 | 0.05 | -0.0003 | 0.05 |
| **cg01671681** | -0.02 | 0.04 | -0.03 | -0.001 |
| **cg07730360** | 0.01 | 0.18 | -0.01 | 0.03 |
| **cg17641710** | -0.002 | 0.80 | -0.01 | 0.01 |
| **cg18098839** | 0.0003 | 0.96 | -0.01 | 0.01 |
| **cg22012981** | 0.005 | 0.41 | -0.01 | 0.02 |
| **cg05119988** | -0.003 | 0.77 | -0.03 | 0.02 |
| **cg06690548** | 0.004 | 0.55 | -0.01 | 0.02 |
| **cg07094298** | -0.003 | 0.78 | -0.03 | 0.02 |
| **cg02286155** | 0.007 | 0.22 | -0.004 | 0.02 |
| **cg04483863** | 0.007 | 0.36 | -0.01 | 0.02 |
| **cg10179300** | 0.008 | 0.14 | -0.003 | 0.02 |
| **cg13276570** | 0.004 | 0.40 | -0.01 | 0.01 |
| **cg13305415** | 0.003 | 0.76 | -0.02 | 0.02 |
| **cg26403843** | -0.02 | 0.08 | -0.04 | 0.002 |
| **cg13123009** | -0.003 | 0.64 | -0.01 | 0.01 |
| **cg14352682** | -0.0003 | 0.96 | -0.01 | 0.01 |
| **cg17501210** | -0.011 | 0.15 | -0.03 | 0.004 |
| **cg17738521** | -0.0001 | 0.99 | -0.01 | 0.01 |
| **cg22875823** | 0.01 | 0.25 | -0.01 | 0.03 |
| **cg04816311** | -0.002 | 0.92 | -0.03 | 0.03 |
| **cg13134297** | -0.008 | 0.45 | -0.03 | 0.01 |
| **cg21429551** | -0.008 | 0.58 | -0.04 | 0.02 |
| **cg02571142** | -0.009 | 0.40 | -0.03 | 0.01 |
| **cg07960624** | -0.02 | 0.19 | -0.04 | 0.01 |
| **cg24531955** | -0.006 | 0.49 | -0.02 | 0.01 |
| **cg25392060** | -0.006 | 0.42 | -0.02 | 0.01 |
| **cg26361535** | 0.01 | 0.24 | -0.01 | 0.03 |
| **cg02716826** | -0.001 | 0.91 | -0.01 | 0.01 |
| **cg07504977** | -0.01 | 0.24 | -0.03 | 0.01 |
| **cg09491962** | -0.004 | 0.53 | -0.02 | 0.01 |
| **cg14333542** | -0.007 | 0.28 | -0.02 | 0.01 |
| **cg15880704** | -0.002 | 0.66 | -0.01 | 0.01 |
| **cg17782974** | -0.007 | 0.50 | -0.03 | 0.01 |
| **cg26955383** | 0.005 | 0.49 | -0.01 | 0.02 |
| **cg00574958** | 0.001 | 0.74 | -0.01 | 0.01 |
| **cg11152384** | -0.007 | 0.18 | -0.02 | 0.003 |
| **cg11261850** | -0.005 | 0.65 | -0.03 | 0.02 |
| **cg13028635** | 0.009 | 0.16 | -0.004 | 0.02 |
| **cg17058475** | 0.0003 | 0.96 | -0.01 | 0.01 |
| **cg26800893** | 0.0005 | 0.90 | -0.01 | 0.01 |
| **cg26894079** | 0.005 | 0.67 | -0.02 | 0.03 |
| **cg13708645** | -0.001 | 0.91 | -0.02 | 0.02 |
| **cg10474597** | 0.004 | 0.69 | -0.01 | 0.02 |
| **cg19750657** | 0.003 | 0.71 | -0.01 | 0.02 |
| **cg10919522** | -0.005 | 0.59 | -0.02 | 0.01 |
| **cg27394566** | 0.004 | 0.53 | -0.01 | 0.02 |
| **cg06192883** | 0.004 | 0.45 | -0.01 | 0.02 |
| **cg07037944** | -0.003 | 0.63 | -0.01 | 0.01 |
| **cg07814318** | 0.001 | 0.91 | -0.02 | 0.02 |
| **cg20507228** | -0.008 | 0.59 | -0.04 | 0.02 |
| **cg21670987** | -0.005 | 0.61 | -0.03 | 0.01 |
| **cg01243823** | -0.02 | 0.02 | -0.04 | -0.003 |
| **cg03500056** | 0.002 | 0.73 | -0.01 | 0.02 |
| **cg06946797** | -0.01 | 0.23 | -0.03 | 0.01 |
| **cg07021906** | 0.02 | 0.12 | -0.004 | 0.03 |
| **cg07955474** | -0.007 | 0.39 | -0.02 | 0.01 |
| **cg09607047** | -0.002 | 0.84 | -0.02 | 0.02 |
| **cg16739178** | 0.002 | 0.83 | -0.02 | 0.02 |
| **cg23813257** | 0.0003 | 0.96 | -0.01 | 0.01 |
| **cg01130991** | 0.001 | 0.93 | -0.02 | 0.02 |
| **cg01597398** | -0.008 | 0.23 | -0.02 | 0.005 |
| **cg01798813** | 0.0002 | 0.97 | -0.01 | 0.01 |
| **cg03078551** | -0.010 | 0.16 | -0.02 | 0.004 |
| **cg04927537** | -0.0005 | 0.96 | -0.02 | 0.02 |
| **cg07012687** | -0.00002 | 1.00 | -0.02 | 0.02 |
| **cg08857797** | 0.01 | 0.23 | -0.01 | 0.03 |
| **cg09664445** | 0.001 | 0.86 | -0.01 | 0.02 |
| **cg10508317** | -0.007 | 0.13 | -0.02 | 0.002 |
| **cg11024682** | -0.004 | 0.39 | -0.01 | 0.01 |
| **cg11202345** | -0.008 | 0.28 | -0.02 | 0.01 |
| **cg13274938** | 0.01 | 0.23 | -0.01 | 0.03 |
| **cg14509967** | 0.009 | 0.24 | -0.01 | 0.02 |
| **cg14870271** | -0.01 | 0.06 | -0.03 | 0.001 |
| **cg16611584** | -0.01 | 0.53 | -0.04 | 0.02 |
| **cg17836612** | -0.001 | 0.90 | -0.02 | 0.01 |
| **cg18091083** | 0.02 | 0.20 | -0.01 | 0.05 |
| **cg18181703** | -0.008 | 0.37 | -0.02 | 0.01 |
| **cg18772573** | 0.01 | 0.19 | -0.01 | 0.03 |
| **cg22713958** | 0.009 | 0.24 | -0.01 | 0.02 |
| **cg25178683** | -0.003 | 0.73 | -0.02 | 0.01 |
| **cg25649826** | 0.001 | 0.87 | -0.01 | 0.01 |
| **cg26651978** | -0.005 | 0.53 | -0.02 | 0.01 |
| **cg27470213** | -0.01 | 0.19 | -0.03 | 0.01 |
| **cg27637521** | -0.005 | 0.17 | -0.01 | 0.002 |
| **cg01751802** | -0.01 | 0.18 | -0.03 | 0.01 |
| **cg02711608** | -0.0001 | 0.99 | -0.01 | 0.01 |
| **cg04557677** | -0.001 | 0.74 | -0.01 | 0.005 |
| **cg07573872** | -0.01 | 0.29 | -0.03 | 0.01 |
| **cg21766592** | -0.01 | 0.07 | -0.02 | 0.001 |
| **cg22950899** | 0.005 | 0.57 | -0.01 | 0.02 |
| **cg26470501** | -0.008 | 0.16 | -0.02 | 0.003 |
| **cg26950531** | 0.005 | 0.73 | -0.02 | 0.03 |
| **cg03218374** | 0.005 | 0.51 | -0.01 | 0.02 |
| **cg00222799** | -0.01 | 0.20 | -0.03 | 0.01 |
| **cg01881899** | -0.004 | 0.34 | -0.01 | 0.004 |
| **cg06500161** | 0.002 | 0.75 | -0.01 | 0.02 |
| **cg10192877** | 0.004 | 0.51 | -0.01 | 0.02 |
| **cg27243685** | -0.001 | 0.90 | -0.02 | 0.01 |
| **cg03682690** | 0.002 | 0.75 | -0.01 | 0.01 |
| **cg06397161** | 0.0002 | 0.97 | -0.01 | 0.01 |
| **cg08548559** | -0.02 | 0.05 | -0.03 | -0.0003 |
| **cg09182678** | -0.006 | 0.25 | -0.02 | 0.004 |
| **cg09349128** | -0.004 | 0.48 | -0.02 | 0.01 |
| **cg14780837** | 0.003 | 0.68 | -0.01 | 0.02 |
| **cg17194270** | 0.004 | 0.71 | -0.02 | 0.03 |
| **cg20496314** | -0.01 | 0.18 | -0.03 | 0.01 |
| **cg22650271** | -0.004 | 0.55 | -0.02 | 0.01 |
| **cg27115863** | -0.01 | 0.16 | -0.03 | 0.005 |

**Supplementary table 4.** Results from the Mendelian Randomisation type analyses for the direction of methylation variation causing BMI.

| **CpG** | **SNP** | **Proxy SNP** | **Beta** | **SE** | **N** | **p-value** |
| --- | --- | --- | --- | --- | --- | --- |
| **cg25392060** | rs12677618 | NA | -0.001 | 0.004 | 235522 | 0.86 |
| **cg01455178** | rs6673687 | NA | 0.01 | 0.004 | 235727 | 0.01 |
| **cg21670987** | rs7172615 | NA | 0.008 | 0.004 | 236092 | 0.05 |
| **cg25649826** | rs7215369 | NA | -0.001 | 0.005 | 230313 | 0.82 |
| **cg21670987** | rs12903325 | NA | 0.008 | 0.004 | 235743 | 0.07 |
| **cg13708645** | rs1549349 | NA | -0.005 | 0.004 | 335771 | 0.22 |
| **cg01751802** | rs11878417 | NA | -0.001 | 0.004 | 235862 | 0.83 |
| **cg07955474** | rs16882 | NA | -0.001 | 0.005 | 234763 | 0.85 |
| **cg23813257** | rs1064948 | NA | -0.005 | 0.004 | 228964 | 0.19 |
| **cg01751802** | rs6511727 | NA | -0.001 | 0.003 | 338998 | 0.71 |
| **cg04286697** | rs2595507 | NA | -0.008 | 0.005 | 235685 | 0.06 |
| **cg22950899** | rs2058110 | NA | -0.002 | 0.005 | 188352 | 0.66 |
| **cg11152384** | rs230539 | NA | -0.003 | 0.004 | 235792 | 0.38 |
| **cg12001357** | rs2099489 | NA | 0.0004 | 0.006 | 179315 | 0.95 |
| **cg14017402** | rs2278536 | NA | -0.006 | 0.004 | 181293 | 0.15 |
| **cg23813257** | rs13335800 | NA | -0.005 | 0.004 | 224535 | 0.18 |
| **cg07955474** | rs11646550 | NA | -0.002 | 0.005 | 228893 | 0.72 |
| **cg03500056** | rs1592457 | NA | 0.003 | 0.004 | 224078 | 0.45 |
| **cg08548559** | rs9621221 | NA | -0.008 | 0.004 | 236207 | 0.03 |
| **cg01751802** | rs7252965 | rs3745682 | 0.001 | 0.004 | 236028 | 0.83 |
| **cg01751802** | rs10421221 | rs4804574 | -0.001 | 0.004 | 236081 | 0.83 |
| **cg04286697** | rs2595503 | rs2290130 | -0.009 | 0.004 | 236094 | 0.04 |
| **cg07955474** | rs1519978 | rs16882 | -0.001 | 0.005 | 234763 | 0.85 |
| **cg08548559** | rs5997938 | rs4820961 | -0.008 | 0.004 | 236196 | 0.03 |
| **cg08548559** | rs230497 | rs230498 | -0.005 | 0.004 | 235825 | 0.17 |
| **cg08548559** | rs8141987 | rs9621216 | -0.008 | 0.004 | 236213 | 0.04 |
| **cg12001357** | rs142273229 | rs2099489 | 0.0004 | 0.006 | 179315 | 0.95 |
| **cg14017402** | rs10178540 | rs2278536 | -0.006 | 0.004 | 181293 | 0.15 |
| **cg24531955** | rs6557652 | rs11135720 | 0.001 | 0.004 | 235962 | 0.70 |
| **cg25649826** | rs4985973 | rs4985974 | -0.002 | 0.005 | 236006 | 0.74 |
| **cg26403843** | rs7732652 | rs1473247 | 0.002 | 0.003 | 339062 | 0.61 |
| **cg26403843** | rs10077858 | rs1897565 | -0.0003 | 0.004 | 236045 | 0.94 |
| **cg26470501** | rs56702353 | rs17728272 | 0.007 | 0.005 | 222495 | 0.14 |

**Supplementary table 5.** Confounders analysis (mother’s smoking and education level) results from 7 different linear models with methylation score as the dependent variable and different combinations of BMI, mother’s smoking and mother’s education as the independent variables, across the 5 time points.

|  |  | **Beta value from Linear model (CI), p-value** | | | | |
| --- | --- | --- | --- | --- | --- | --- |
| **Linear Model** |  | **Birth** | **Childhood** | **Adolescence** | **Pregnancy** | **Middle-age** |
| **Methylation ~ BMI** | BMI | 0.0003 (0.0002, 0.0005), 9.79E-06^a,b^ | 0.05 (0.01, 0.08), 0.005^a,b^ | 0.05 (0.03, 0.07), 1.05E-06^a,b^ | 0.04 (0.02, 0.06), 2.72E-05^a,b^ | 0.06 (0.05, 0.07), 2.15E-21^a,b^ |
| **Methylation ~ BMI + Mother smokes** | BMI | 0.0003 (0.0001, 0.0004), 0.0001^a,b^ | 0.05 (0.01, 0.08), 0.008^a^ | 0.05 (0.03, 0.07), 3.46E-06^a,b^ | 0.04 (0.02, 0.07), 5.92E-04^a,b^ | 0.06 (0.04, 0.07), 132E-14^a,b^ |
|  | Mother smokes | -0.18 (-0.35, -0.007), 0.04^a^ | -0.001 (-0.17, 0.17), 0.99 | 0.05 (-0.13, 0.24), 0.57 | 0.03 (-0.19, 0.25), 0.79 | 0.11 (-0.10, 0.31), 0.30 |
| **Methylation ~ Mother smokes** | Mother smokes | -0.20 (-0.37, -0.02), 0.03^a^ | 0.02 (-0.15, 0.18), 0.83 | 0.09 (-0.09, 0.27), 0.34 | 0.07 (-0.16, 0.29), 0.55 | 0.15 (-0.06, 0.37), 0.16 |
| **Methylation ~ BMI + Education of mother** | BMI | 0.0003 (0.0002, 0.0005), 2.51E-05^a,b^ | 0.05 (0.01, 0.08), 0.005^a,b^ | 0.05 (0.03, 0.07), 1.70E-06^a,b^ | 0.04 (0.02, 0.06), 4.92E-05^a,b^ | 0.06 (0.05, 0.07), 4.94E-20^a,b^ |
|  | Education (Vocational) | -0.03 (-0.39, 0.33), 0.88 | 0.15 (-0.21, 0.52), 0.42 | 0.15 (-0.24, 0.53), 0.45 | 0.09 (-0.29, 0.47), 0.64 | 0.32 (-06, 0.69), 0.10 |
|  | Education (O-level) | 0.05 (-0.24, 0.34), 0.76 | -0.009 (-0.30, 0.28), 0.95 | 0.11 (-0.20, 0.43), 0.48 | 0.15 (-0.16, 0.46), 0.34 | 0.14 (-0.17, 0.45), 0.37 |
|  | Education (A-level) | 0.12 (-0.18, 0.41), 0.44 | 0.003 (-0.29, 0.30), 0.98 | 0.09 (-0.23, 0.41), 0.58 | 0.04 (-0.27, 0.35), 0.78 | 0.16 (-0.15, 0.47), 0.32 |
|  | Education (Degree) | 0.08 (-0.23, 0.38), 0.62 | 0.12 (-0.19, 0.42), 0.45 | 0.03 (-0.29, 0.36), 0.84 | 0.14 (-0.19, 0.46), 0.41 | 0.18 (-0.14, 0.51), 0.28 |
| **Methylation ~ BMI + Mother smokes + Education of mother** | BMI | 0.0003 (0.0001, 0.0004), 0.0002^a,b^ | 0.05 (0.01, 0.08), 0.009^a^ | 0.05 (0.03, 0.07), 4.15E-06^a,b^ | 0.04 (0.02, 0.07), 5.31E-04^a,b^ | 0.06 (0.04, 0.07), 1.52E-14^a,b^ |
|  | Mother smokes | -0.14 (-0.32, 0.04), 0.12 | 0.03 (0.01, 0.08), 0.73 | 0.07 (-0.12, 0.26), 0.46 | 0.02 (-0.22, 0.25), 0.89 | 0.11 (-0.10, 0.32), 0.29 |
|  | Education (Vocational) | -0.05 (-0.42, 0.33), 0.80 | 0.18 (-0.20, 0.55), 0.36 | 0.18 (-0.22, 0.58), 0.39 | 0.24 (-0.27, 0.75), 0.36 | 0.33 (-0.15, 0.80), 0.18 |
|  | Education (O-level) | -0.006 (-0.31, 0.30), 0.97 | -0.03 (-0.33, 0.28), 0.87 | 0.14 (-0.19, 0.47), 0.40 | 0.21 (-0.20, 0.61), 0.32 | 0.18 (-0.21, 0.56), 0.38 |
|  | Education (A-level) | 0.07 (-0.24, 0.38), 0.66 | 0.02 (-0.28, 0.33), 0.88 | 0.12 (-0.21, 0.46), 0.47 | 0.10 (-0.31, 0.50), 0.64 | 0.27 (-0.12, 0.66), 0.17 |
|  | Education (Degree) | 0.04 (-0.29, 0.36), 0.82 | 0.14 (-0.18, 0.46), 0.39 | 0.06 (-0.28, 0.41), 0.72 | 0.20 (-0.22, 0.61), 0.35 | 0.31 (-0.09, 0.71), 0.14 |
| **Methylation ~ Education of mother** | Education (Vocational) | -0.02 (-0.39, 0.34), 0.91 | 0.16 (-0.20, 0.53), 0.39 | 0.13 (-0.26, 0.52), 0.52 | 0.12 (-0.27, 0.50), 0.55 | 0.28 (-0.12, 0.68), 0.17 |
|  | Education (O-level) | 0.07 (-0.39, 0.34), 0.66 | -0.009 (-0.30, 0.28), 0.95 | 0.12 (-0.20, 0.44), 0.47 | 0.13 (-0.18, 0.44), 0.41 | 0.01 (-0.31, 0.34), 0.93 |
|  | Education (A-level) | 0.13 (-0.17, 0.43), 0.40 | 0.002 (-0.30, 0.28), 0.99 | 0.06 (-0.26, 0.38), 0.71 | 0.01 (-0.30, 0.33), 0.70 | 0.03 (-0.30, 0.36), 0.86 |
|  | Education (Degree) | 0.10 (-0.21, 0.41), 0.51 | 0.10 (-0.20, 0.40), 0.51 | -0.02 (-0.35, 0.31), 0.90 | 0.07 (-0.25, 0.39), 0.67 | -0.05 (-0.39, 0.29), 0.76 |
| **Methylation ~ Education of mother + Mother smokes** | Mother smokes | -0.15 (-0.34, 0.03), 0.09 | 0.05 (-0.12, 0.22), 0.58 | 0.10 (-0.09, 0.29), 0.29 | 0.05 (-0.18, 0.28), 0.68 | 0.14 (-0.08, 0.37), 0.20 |
|  | Education (Vocational) | -0.04 (-0.42, 0.34), 0.83 | 0.19 (-0.19, 0.57), 0.33 | 0.16 (-0.24, 0.57), 0.43 | 0.25 (-0.26, 0.76), 0.34 | 0.31 (-0.20, 0.81), 0.23 |
|  | Education (O-level) | 0.01 (-0.30, 0.32), 0.95 | -0.03 (-0.33, 0.28), 0.87 | 0.15 (-0.18, 0.48), 0.43 | 0.18 (-0.22, 0.59), 0.38 | 0.06 (-0.35, 0.47), 0.77 |
|  | Education (A-level) | 0.08 (-0.23, 0.39), 0.62 | 0.02 (-0.28, 0.33), 0.88 | 0.10 (-0.23, 0.44), 0.55 | 0.08 (-0.33, 0.49), 0.70 | 0.18 (-0.23, 0.59), 0.39 |
|  | Education (Degree) | 0.06 (-0.27, 0.38), 0.72 | 0.13 (-0.19, 0.45), 0.43 | 0.02 (-0.33, 0.37), 0.91 | 0.13 (-0.29, 0.55), 0.53 | 0.11 (-0.31, 0.53), 0.61 |

a= significant at the 0.05 p-value threshold, b= significant at the adjusted p-value threshold of 0.007 (0.05/7, as there were 7 models).

**Supplementary table 6.** Results from ANOVA tests comparing the model adjusting for methylation score and BMI (outcome ~ BMI + methylation score) with the model with just BMI (outcome ~ BMI) in the first columns and then the full model (outcome ~ methylation score + genetic score) is compared with the model with just methylation score (outcome ~ methylation score) in the other columns. These tests were additionally conducted for the models with and without adjustment for BMI, as indicated in the table. The data used was the adolescent data for children and the middle-aged data for mothers.

|  | **Child (adolescence)** | | | | | | | **Mother (middle age)** | | | | | | |
| --- | --- | --- | --- | --- | --- | --- | --- | --- | --- | --- | --- | --- | --- | --- |
|  | **Comparing:**  **1.** **outcome ~ BMI**  **2. outcome ~ BMI + methylation score** | | **Comparing:**  **1. outcome ~ methylation score**  **2. outcome ~ methylation score + genetic score** | | **Comparing:**  **1. outcome ~ methylation score + BMI**  **2. outcome ~ methylation score + genetic score + BMI** | | **Ratio of DNAm R^2^:BMI R^2^** | **Comparing:**  **1.** **outcome ~ BMI**  **2. outcome ~ BMI + methylation score** | | **Comparing:**  **1. outcome ~ methylation score**  **2. outcome ~ methylation score + genetic score** | | **Comparing:**  **1. outcome ~ methylation score + BMI**  **2. outcome ~ methylation score + genetic score + BMI** | | **Ratio of DNAm R^2^:BMI R^2^** |
|  | **F** | **p-value** | **F** | **p-value** | **F** | **p-value** |  | **F** | **p-value** | **F** | **p-value** | **F** | **p-value** |  |
| **log (triglycerides)** | 1.44 | 0.18 | 0.03 | 0.88 | 1.42 | 0.23 | 1:18 | 2.81 | 3.00E-03 | 1.69 | 0.19 | 8.71 | 3.27E-03 | 1:6 |
| **LDL** | 2.50 | 0.02 | 0.48 | 0.49 | 0.19 | 0.67 | 1:1 | 2.36 | 0.01 | 0.09 | 0.76 | 0.50 | 0.48 | 1:3 |
| **log(glucose)** | 2.20 | 0.03 | 0.65 | 0.42 | 0.01 | 0.92 | 1:3.33 | 1.73 | 0.08 | 5.05 | 0.02 | 1.71 | 0.19 | 1:5.56 |
| **log(insulin)** | 0.84 | 0.56 | 0.07 | 0.79 | 2.80 | 0.09 | 1:32.5 | 1.52 | 0.14 | 0.64 | 0.43 | 1.92 | 0.17 | 1:54 |
| **SBP** | 1.85 | 0.08 | 16.11 | 6.60E-05 | 3.78 | 0.05 | 1:50 | 2.11 | 0.03 | 0.04 | 0.83 | 2.36 | 0.13 | 0.004: 0.14 |
| **DBP** | 1.14 | 0.33 | 4.63 | 0.03 | 0.17 | 0.68 | 1:8.75 | 1.44 | 0.17 | 1.45 | 0.23 | 0.24 | 0.62 | 1:25 |

**Supplementary table 7.** Results from ANOVA tests comparing the model adjusting for genetic score and BMI (outcome ~ BMI + genetic score) with the model with just BMI (outcome ~ BMI) in the first columns and then the full model (outcome ~ methylation score + genetic score) with the model with just genetic score (outcome ~ genetic score) in the other columns. These tests were additionally conducted for the models with and without adjustment for BMI, as indicated in the table. The data used was the adolescent data for children and the middle-aged data for mothers.

|  | **Child** | | | | | | **Mother** | | | | | |
| --- | --- | --- | --- | --- | --- | --- | --- | --- | --- | --- | --- | --- |
|  | **Comparing:**  **1.** **outcome ~ BMI**  **2. outcome ~ BMI + genetic score** | | **Comparing:**  **1. outcome ~ genetic score**  **2. outcome ~ methylation score + genetic score** | | **Comparing:**  **1. outcome ~ genetic score + BMI**  **2. outcome ~ methylation score + genetic score + BMI** | | **Comparing:**  **1.** **outcome ~ BMI**  **2. outcome ~ BMI + genetic score** | | **Comparing:**  **1. outcome ~ genetic score**  **2. outcome ~ methylation score + genetic score** | | **Comparing:**  **1. outcome ~ genetic score + BMI**  **2. outcome ~ methylation score + genetic score + BMI** | |
|  | **F** | **p-value** | **F** | **p-value** | **F** | **p-value** | **F** | **p-value** | **F** | **p-value** | **F** | **p-value** |
| **log(triglycerides)** | 1.44 | 0.18 | 2.93 | 5.00E-03 | 1.47 | 0.18 | 3.43 | 2.01E-04 | 7.21 | 4.66E-10 | 2.74 | 3.80E-03 |
| **LDL** | 2.21 | 0.03 | 2.80 | 7.00E-03 | 2.51 | 0.02 | 2.17 | 0.02 | 3.41 | 4.10E-04 | 2.34 | 0.01 |
| **log(glucose)** | 1.92 | 0.05 | 1.89 | 0.07 | 2.18 | 0.03 | 1.73 | 0.07 | 2.31 | 0.01 | 1.71 | 0.08 |
| **log(insulin)** | 1.08 | 0.37 | 2.44 | 0.02 | 0.83 | 0.56 | 1.55 | 0.12 | 6.92 | 1.39E-09 | 1.51 | 0.14 |
| **SBP** | 2.10 | 0.03 | 2.90 | 5.38E-03 | 1,71 | 0.10 | 2.14 | 0.02 | 3.45 | 3.51E-04 | 2.17 | 0.02 |
| **DBP** | 1.02 | 0.42 | 2.15 | 0.04 | 1.14 | 0.34 | 1.32 | 0.22 | 2.01 | 0.04 | 1.46 | 0.16 |

**Supplementary figure 1.** QQ plot of the p-values from the association between each of the 135 CpG sites and BMI in the middle-aged mothers.


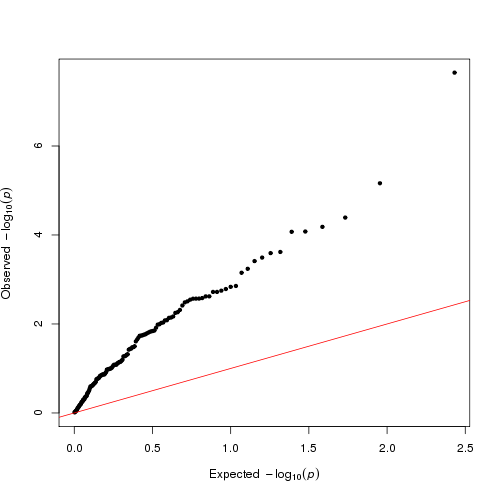


**Supplementary figure 2.** QQ plot of the p-values from the association between each of the 135 CpG sites and BMI in the adolescent children.

**
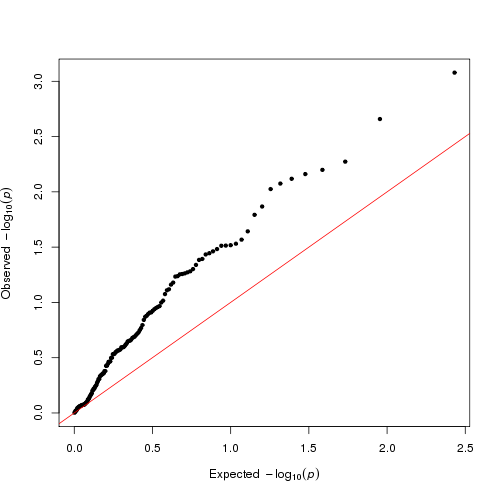
**

**Supplementary figure 3.** Scatterplot of effect sizes from the *Mendelson et al* paper against those in ARIES in the middle-aged mothers.


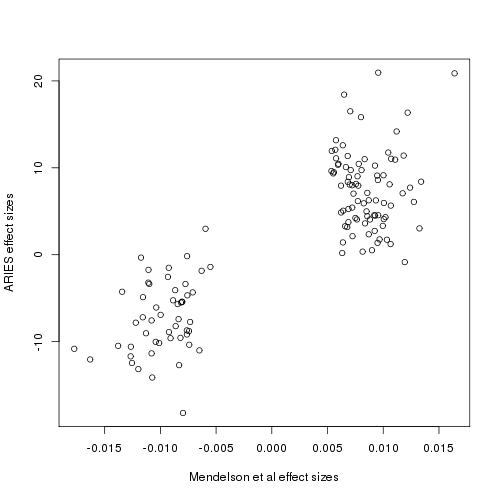


**Supplementary figure 4.** Scatterplot of effect sizes from the *Mendelson et al* paper against those in ARIES in the adolescent children.

**
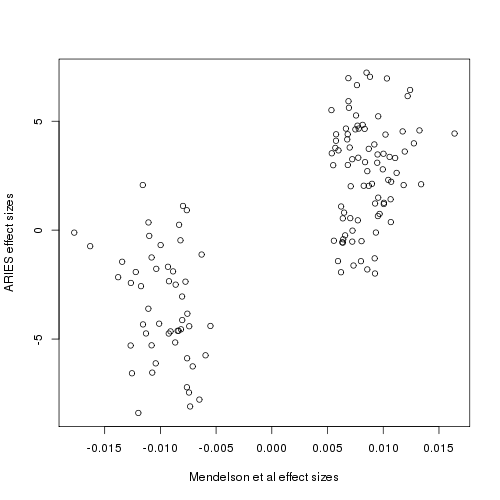
**

**Supplementary figure 5.** Cross-lagged model for sub model with path from childhood DNA methylation to adolescent BMI dropped, in children.

**
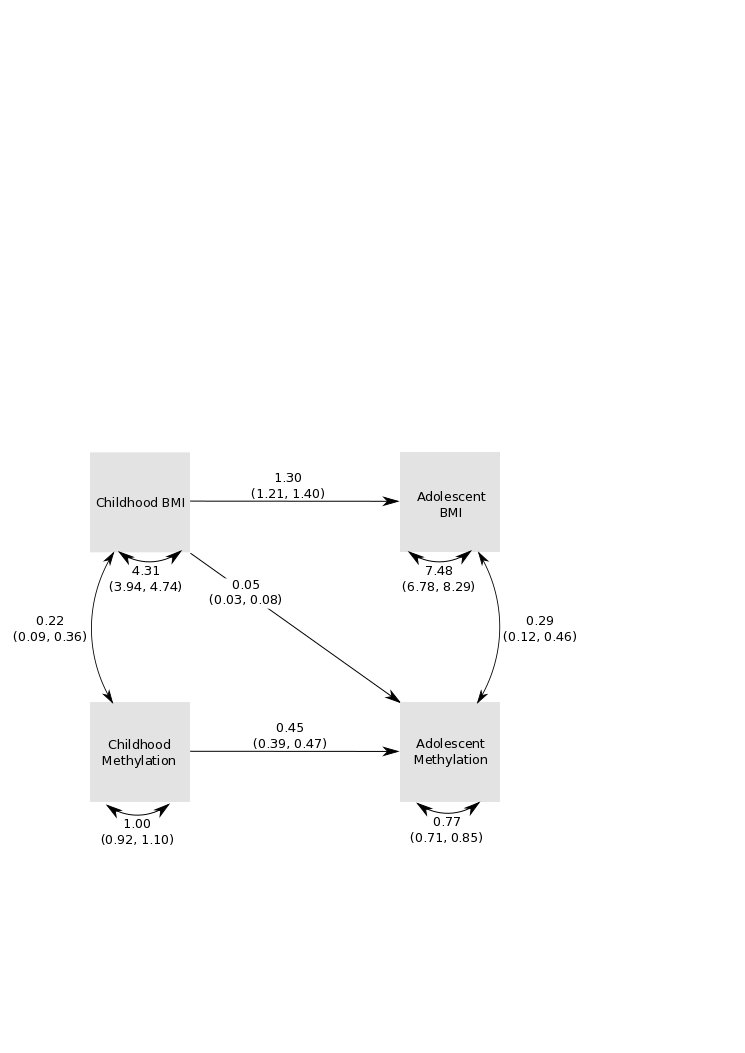
**

**Supplementary figure 6.** Cross-lagged model for sub model path from DNA methylation score in pregnancy to BMI in middle-age dropped, in mothers.


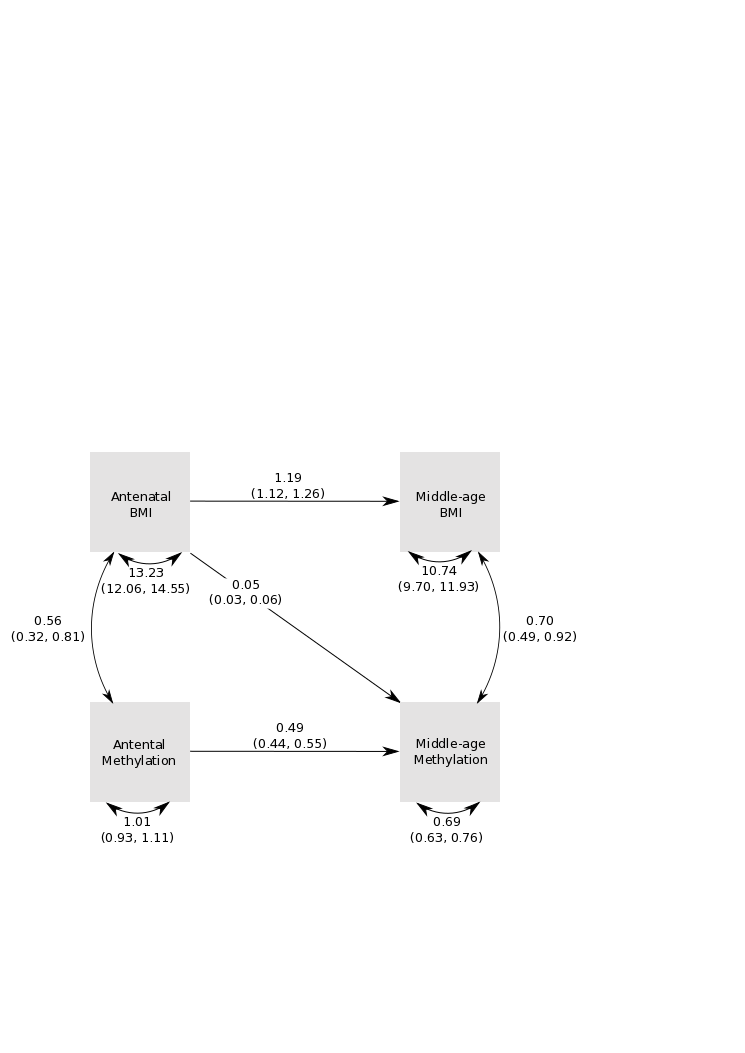

Supplement: Supplementary file 1 — Additional file 1: This contains supplementary tables S1-S7 and supplementary figures S1-S2. [file 13148_2020_841_MOESM1_ESM.docx]
